# Detection of Zoonotic Gastrointestinal Pathogens in Dairy Sheep and Goats by Using FilmArray^®^ Multiplex-PCR Technology

**DOI:** 10.3390/microorganisms10040714

**Published:** 2022-03-25

**Authors:** Katerina Tsilipounidaki, Zoe Florou, Daphne T. Lianou, Charalambia K. Michael, Eleni I. Katsarou, Anargyros Skoulakis, George C. Fthenakis, Efthymia Petinaki

**Affiliations:** 1 University Hospital of Larissa, 41110 Larissa, Greece; tsilipoukat@gmail.com (K.T.); zoi_fl@yahoo.gr (Z.F.); skulakis@gmail.com (A.S.); petinaki@med.uth.gr (E.P.); 2 Veterinary Faculty, University of Thessaly, 43100 Karditsa, Greece; dlianou@vet.uth.gr (D.T.L.); cmichail@vet.uth.gr (C.K.M.); elekatsarou@vet.uth.gr (E.I.K.)

**Keywords:** *Campylobacter*, *Cryptosporidium*, diarrhoea, *Escherichia coli*, FilmArray, *Giardia*, goat, sheep, Rotavirus, *Salmonella*, *Vibrio*, *Yersinia*, zoonoses

## Abstract

The objectives of this study were (a) to detect gastrointestinal pathogens in faecal samples of sheep and goats using the FilmArray^®^ GI Panel and (b) to evaluate factors that were associated with their presence. Faecal samples from ewes or does in 70 sheep flocks and 24 goat herds in Greece were tested for the presence of 22 gastrointestinal pathogens by means of the BioFire^®^ FilmArray^®^ Gastrointestinal (GI) Panel. The most frequently detected pathogens were Shiga-like toxin-producing *Escherichia coli* stx1/stx2 (94.7% of farms), *Giardia lamblia* (59.6%), and *Campylobacter* spp. (50.0% of farms). Other pathogens detected were *Cryptosporidium* spp., *Salmonella* spp., enterotoxigenic *E. coli* lt/st, *Yersinia enterocolitica*, *E. coli* O157, Rotavirus A, *Shigella*/enteroinvasive *E. coli,* and *Plesiomonas* *shigelloides*. There was a difference in the prevalence of detection of pathogens between sheep and goat farms only for *Salmonella* spp.: 18.3% versus 0.0%, respectively. Mixed infections were detected in 76 farms (80.9% of farms), specifically 57 sheep flocks and 19 goat herds, with on average, 2.5 ± 0.1 pathogens detected per farm. The body condition score of ewes in farms, in which only one pathogen was detected in faecal samples, was significantly higher than that of ewes in farms, in which at least two pathogens were detected: 2.55 ± 0.11 versus 2.31 ± 0.04. In sheep flocks, the number of pathogens in faecal samples was significantly higher in farms with semi-extensive management. In goat herds, the number of pathogens in faecal samples was positively correlated with average precipitation and inversely correlated with temperature range in the respective locations.

## 1. Introduction

Gastrointestinal infections are widely recognized health problems of animals and people, caused by a variety of bacteria, protozoa, metazoa, or viruses. The causal pathogens are often disseminated from animals to humans through food products or direct contact.

Adult dairy small ruminants are particularly prone to gastrointestinal infections as a result of grazing. Adult sheep and goats can be carriers of pathogens, which they may transmit to newborns, during the post-partum period, when pathogen shedding is increased as a consequence of the relaxation of immunity, which occurs during the peri-parturient period [1,2]. Lambs and kids may become infected while sucking the dirty udder of their dams or licking the infected bedding or ground on the farms [3]. Moreover, pathogens can contaminate raw milk, for example during milking, particularly when adequate hygiene procedures are not followed, which poses a threat for subsequent zoonotic infections of consumers.

Clinical diagnosis is difficult and often inconclusive, as relevant clinical findings are in general similar across the various infections; moreover, often the infections remain subclinical. Definitive diagnosis can be achieved only by means of detailed laboratory examinations of faecal samples. Testing requires time, which would delay the initiation of the appropriate treatment course, if necessary. Moreover, some of the techniques most often applied have low accuracy [4,5]. Therefore, rapid and reliable tests are required to diagnose gastrointestinal infections of small ruminants, which would support decisions for early intervention [6].

Presently, numerous rapid detection methods are commercially available for the detection of gastrointestinal pathogens, such as immunoassays or nucleic acid amplification methods. The ‘BioFire^®^ FilmArray^®^ Gastrointestinal (GI) Panel’ (BioFire Diagnostics, Salt Lake City, UT, USA) is a fully automated multiplexed nucleic acid-based test for the qualitative in vitro detection and identification of multiple pathogens. The panel can detect simultaneously 22 different gastrointestinal pathogens (13 bacteria, 4 protozoa, 5 viruses) and was developed primarily for the rapid detection and identification of a multitude of pathogens in clinical samples from people [7]. However, the panel was found to have high accuracy for detecting pathogens in other matrixes as well, e.g., water [8]. In major validation studies, the overall specificity of the assay was found to be >97% [7,9,10], while the overall reproducibility and negative percent agreement are considered to >99% [11].

There is scope for monitoring the gastrointestinal pathogens in sheep and goats in Greece, because of the significance of these animal species for the agricultural economy of the country [12]. The objectives of this study were (a) to detect zoonotic gastrointestinal pathogens in faecal samples of sheep and goats using the FilmArray^®^ GI Panel and (b) to evaluate factors that were associated with their presence.

## 2. Materials and Methods

### 2.1. Farms and Samplings

A cross-sectional study involving 70 sheep flocks and 24 goat herds was performed from November 2019 to March 2020 in Greece. Farms were included into the study on a per convenience basis. Farms were located in the administrative regions of Thessaly (*n* = 48 and 11, respectively), Peloponnese (*n* = 17 and 7), Western Greece (*n* = 6 and 3), Epirus (*n* = 1 and 0) and Ionian Islands (*n* = 1 and 0). Farms were included in the study on a per convenience basis (willingness of farmers to receive a visit from university staff), as described previously [13,14,15]. Three of the investigators (authors C.K.M., D.T.L., and G.C.F.) visited all the farms for sample collection. The management practices applied in the flocks and herds were recorded during an interview of the farmer by means of a detailed questionnaire [13]. Questions were related to infrastructure, animals, production characteristics, health management, nutrition, and human resources on the farm [13]. The management system applied on the farms (intensive, semi-intensive, semi-extensive, extensive) was defined according to the relevant classification by the European Food Safety Authority [16].

Faecal samples were collected from the rectum of ewes or does on the farm. On each farm, 20, 30, 40, or 50 females in the milking period (for farms with 165, 166–330, 331–500 or >500 females, respectively) were selected for sampling. For selection of animals for sampling, these were walked into the milking area and the necessary number of females was selected as the animals walked in, using an electronic random number generator (www.randomresult.com), and thereafter sampled. Approximately 5 g of each of the individual animal faecal samples from a farm were taken and mixed to form a pooled faecal sample of the animals on the farm. The pooled sample was homogenized by mild bead-beating [17] and swab samples were taken and immersed into Cary Blair transport media.

Ewes and does were also evaluated for body condition scoring. To ensure uniformity of measurements and adherence to published standards [18], scoring (0–5, including half scores) was always carried out by a certified European Veterinary Specialist in Small Ruminant Health Management.

Faecal samples were stored at 8.0 to 10.0 °C using portable refrigerators. Transportation of samples to the laboratory was made by the investigators and by car; samples collected from flocks and herds on the islands were also transported as accompanying luggage by airplane (Crete, Lesvos. and Rhodes) or by boat (Cephalonia).

### 2.2. Laboratory Examinations

Laboratory examinations started within 16 to 24 h after collection of samples. All the pooled faecal samples were tested by means of the BioFire^®^ FilmArray^®^ Gastrointestinal (GI) Panel. The panel has the capacity to detect genetic material of the following microorganisms: *Campylobacter* (*jejuni*, *coli*, *upsaliensis*), *Clostridium difficile* (toxin A/B), *Plesiomonas shigelloides*, *Salmonella* spp., *Yersinia enterocolitica*, *Vibrio* (*parahaemolyticus*, *vulnificus*, *cholerae*), *Vibrio cholerae*, enteroaggregative *Escherichia coli*, enteropathogenic *E. coli*, enterotoxigenic *E. coli* lt/st, Shiga-like toxin-producing *E. coli* stx1/stx2, *E. coli* O157, *Shigella*/enteroinvasive *E. coli*, *Cryptosporidium*, *Cyclospora cayetanensis*, *Entamoeba histolytica*, *Giardia lamblia*, Adenovirus F40/41, Astrovirus, Norovirus GI/GII, Rotavirus A and Sapovirus (I, II, IV, and V).

A quantity of 200 μL of the sample was added to each panel according to the manufacturer’s instructions [19], for analysis in The BioFire^®^ FilmArray^®^ 2.0 System (BioFire Diagnostics). The actual test was completed within one hour.

### 2.3. Data Management and Analysis

Data were entered into Microsoft Excel and analyzed using SPSS v. 21 (IBM Analyt-ics, Armonk, NY, USA). A basic descriptive analysis was performed and exact binomial confidence intervals (CIs) were obtained.

As part of a wider study of parasites of small ruminants in Greece, a standardized examination of faecal samples was performed using conventional parasitological techniques for detection of *Cryptosporidium* spp. and *Giardia* spp. [20]. The proportions of samples in which these two protozoan pathogens were detected by each of the methodologies (conventional parasitological technique and FilmArray^®^ technology) were compared by means of the two-sample z test of proportions.

The potential association of the total number of pathogens detected in faecal samples from a farm with the mean body condition score of animals evaluated in each flock/herd was assessed using analysis of variance. Additionally, the potential association of the total number of pathogens detected in faecal samples from a farm with the average annual milk production per ewe/doe on the farm, as deduced from the answers of the farmer during the interview [13], was also assessed by analysis of variance.

In total, four management-related variables (management system applied on the farm, one month into the lactation period at sampling, availability of milking parlour, number of female animals in the flock/herd) were evaluated for potential association with the presence of pathogens in pooled faecal samples; these were either taken from the answers of the interview performed at the start of the visit. For each of these variables, categories were created according to the answers of the farmers.

The outcomes of “detection of a specific pathogen in faecal samples” (specifically, *Campulobacter* spp., *Salmonella* spp., *Y. enterocolitica*, enterotoxigenic *E. coli* lt/st, Shiga-like toxin-producing *E. coli* stx1/stx2, *E. coli* O157, *Shigella*/enteroinvasive *E. coli*, *Cryptosporidium* spp., *G. lamblia* and Rotavirus A, i.e., in total 10 different outcomes) were considered. Exact binomial CI were obtained. The importance of predictors was assessed using cross-tabulation with Pearson’s chi-square test. Separate analyses were performed for sheep flocks and goat herds. Then, the outcome “detection of mixed infection in faecal samples” was considered. The importance of predictors was assessed using analysis of variance. Again, separate analyses were performed for sheep flocks and goat herds.

During the visit on each farm, data on farm location were collected using hand-held Global Positioning System Garmin units. The geo-references were resolved to the specific farm level.

Climatic variables were derived from ‘The POWER (Prediction of Worldwide Energy Resources) Project’ (NASA Langley Research Center (LaRC), Hampton, VA, USA), which provides meteorological datasets from NASA research for the support of agricultural needs. The following settings were used for obtaining the data: user community, ‘*agroclimatology*’; temporal average, ‘*monthly & annual*’; latitude/longitude, ‘*geo-references of each farm*’; time extent, ‘*start date 15 days prior to the date of the visit–end date one day prior to the date of the visit*’; output file format, ‘*ASCII*’. Data for the following parameters were extracted: temperature at 2 m (T2M), temperature of Earth skin (TS), minimum temperature at 2 m (T2Min), maximum temperature at 2 m (T2Max), temperature range at 2 m (T2Ran), precipitation (PREC), and all sky insolation incident (INSOL). For the evaluations, the averages for the 15 days prior to the visit, as provided by the above platform for the location of each farm, were taken into account. The potential association of the number of pathogens detected in faecal samples with the climatic results for the location of the farm was initially assessed using analysis of correlation. Then, farms were allocated in one of three cohorts, in which 1 or 2 or ≥3 pathogens were detected in the respective samples; climatic parameters were compared between the locations of the farms in the three cohorts using analysis of variance.

In all analyses, statistical significance was defined at *p* ≤ 0.05.

## 3. Results

### 3.1. Pathogens Detected in Faecal Samples

In all faecal samples tested (100.0% (96.1–100.0%)), at least one gastrointestinal pathogen was detected. The most frequently detected pathogens were Shiga-like toxin-producing *E. coli* stx1/stx2 (94.7% of farms), *G. lamblia* (59.6%) and *Campylobacter* spp. (50.0% of farms). Other pathogens detected were *Cryptosporidium* spp. (14.9% of farms), *Salmonella* spp. and enterotoxigenic *E. coli* lt/st (each from 13.8% of farms), *Y. enterocolitica* (11.7%), *E. coli* O157 (10.6%), Rotavirus A (5.3%), *Shigella*/enteroinvasive *E. coli* (4.3%), and *P. shigelloides* (1.1% of farms).

There was a significant difference in the prevalence of detection of pathogens between sheep and goat farms only for *Salmonella* spp.: 18.3% versus 0.0% respectively (*p* = 0.022). For the difference in the prevalence of detection of the other pathogens between sheep and goat farms, no such significance was seen (*p* ≥ 0.07 for all comparisons). Details of the prevalence of the individual pathogens in the sampled farms are shown in Table 1.

The proportion of faecal samples in which *Giardia* spp. or *Cryptosporidium* spp. were detecte, was higher using the FilmArray^®^ technology than the conventional parasitological techniques: 59.6% and 33.0% [20], versus 14.9% and 6.4% [20], respectively (*p* = 0.0001 and *p* = 0.029, respectively).

### 3.2. Detection of More Than One Pathogen in Faecal Samples

Mixed infections were detected in 76 farms, specifically in 57 sheep flocks (81.4% (70.8–88.8%)) and 19 goat herds (79.2% (59.5–90.8%)) (*p* = 0.81). On average, 2.5 ± 0.1 pathogens were detected per farm; no difference was evident between sheep flocks and goat herds: 2.6 ± 0.1 versus 2.3 ± 0.2 pathogens per farm (*p* = 0.25).

The two most commonly detected combinations of pathogens were Shiga-like toxin-producing *E. coli* stx1/stx2 and *G. lamblia* (57.1% of sheep flocks, 45.8% of goat farms) and Shiga-like toxin-producing *E. coli* stx1/stx2 and *Campylobacter* spp. (51.4% of sheep flocks, 37.5% of goat herds). Moreover, a triple mixed infection (Shiga-like toxin-producing *E. coli* stx1/stx2, *G. lamblia* and *Campylobacter* spp.) (37.1% of sheep flocks, 20.8% of goat herds) was also seen often. There was no difference in the frequency of the various combinations of infections between sheep and goat farms (*p* > 0.14 for all comparisons).

### 3.3. Association of Detection of Pathogens with Clinical Findings

The mean body condition score of ewes in farms, in which only one pathogen was detected in faecal samples, was significantly higher than that of ewes in farms, in which at least two pathogens were detected: 2.55 ± 0.11 versus 2.31 ± 0.04, respectively (*p* = 0.014). In contrast, no such difference was seen for does: 2.61 ± 0.06 versus 2.69 ± 0.61, respectively (*p* = 0.13).

Νo significant differences were seen in the average annual milk production of ewes and does, when classified in accord with the detection of one or more pathogens in faecal samples: 242 ± 24 mL versus 208 ± 10 mL for sheep and 252 ± 50 mL versus 186 ± 42 mL for goats, respectively (*p* > 0.16 for all comparisons).

### 3.4. Association of Detection of Pathogens with Management-Related Variables

On sheep farms, associations were seen in the detection of *Y. enterocolitica* with the management system applied in farms (*p* = 0.033) and the availability of milking parlour (*p* = 0.002) and for the detection of enterotoxigenic *E. coli* lt/st with the availability of milking parlour (*p* = 0.046) (Table 2). For that latter pathogen, the same association (i.e., with the availability of milking parlour) was seen in goat farms (*p* = 0.020) (Table 2). No other associations were found. The detailed results of the univariable analyses for potential associations with the detection of a pathogen are in Tables S1 (sheep) and S2 (goats).

On sheep farms, an association was seen for the number of pathogens detected in faecal samples with the management system applied therein; in flocks with intensive, semi-intensive or semi-extensive management 2.3 ± 0.5, 2.3 ± 0.4 and 3.2 ± 0.3 pathogens were detected in faecal samples (*p* = 0.010). No other associations were found in sheep flocks or in goat herds (Table S3).

### 3.5. Association of Detection of Pathogens with Climatic Factors

On sheep farms, no correlations were seen between the number of pathogens detected in faecal samples and the average for the 15 days prior to the visit for any climatic parameter (|*r*| < 0.15, *p* > 0.10 for all comparisons). Moreover, no significant differences were evident between farms in the faecal samples from which 1 or 2 or ≥3 pathogens were detected (*p* > 0.20 for all comparisons) (Table S4).

**Figure 1:**
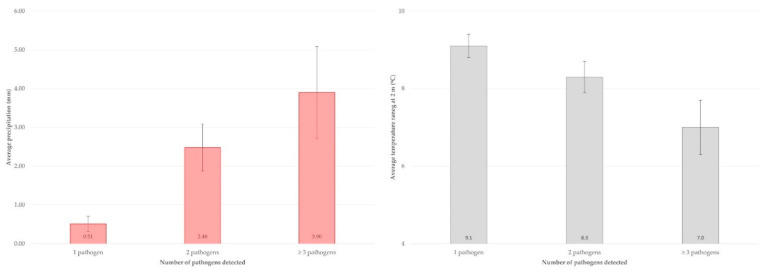
Average precipitation (**left** histogram) and average temperature at 2 m (**right** histogram) in the locations of goat farms for the 15 days prior to sampling in accord with the number of pathogens recovered in faecal samples from these farms.

In contrast, on goat farms, there was evidence of correlation between the number of pathogens detected in faecal samples and the average for the 15 days prior to the visit for some climatic parameters, as follows: average minimum temperature at 2 m (*r* = 0.3841, *p* = 0.032), average temperature range at 2 m (*r* = −0.5133, *p* = 0.005), average precipitation (*r* = 0.5123, *p* = 0.005) and average all sky insolation incident (*r* = −0.3960, *p* = 0.028) (for the other climatic parameters assessed: |*r*| < 0.34, *p* > 0.05 for all comparisons). Moreover, a significant difference was seen between farms, in the faecal samples from which 1 or 2 or ≥3 pathogens were detected, for average temperature range at 2 m (9.1 ± 0.3 °C, 8.3 ± 0.4 °C, 7.0 ± 0.7 °C, respectively; *p* = 0.05) and average precipitation (0.51 ± 0.20 mm, 2.48 ± 0.60 mm, 3.90 ± 1.18 mm, respectively; *p* = 0.044), while a trend also emerged for the average all sky insolation incident (10.6 ± 0.4 Wh m^−2^, 9.1 ± 0.5 Wh m^−2^, 7.5 ± 0.6 Wh m^−2^, respectively; *p* = 0.07) (for the other climatic parameters assessed: *p* > 0.18 for all comparisons) (Figure 1, Table S4).

## 4. Discussion

### 4.1. The Use of FilmArray^®^ GI Panel Technology in Samples from Small Ruminants

FilmArray^®^ GI Panel employs multiplex-PCR technology and is used to detect the most common enteropathogens of people, directly from faecal samples. The procedure takes approximately one hour, which leads to a quick diagnosis. The method received approval from the Food and Drug Administration in 2014 [11] and since then has been used in quick and reliable diagnosis of gastroinstestinal infections in people.

The technology has not been previously employed on animal samples. To the best of our knowledge, there is only one previous report of using the technology to analyze faecal samples from animals, which was performed in Spain with 45 faecal samples from hedgehogs [21]. In the present study, the method was found to be useful and provided the results in a convenient manner. Nevertheless, as the technology is oriented for use on human samples, a limitation was the impossibility to detect pathogens of mainly veterinary interest, for example, *Mycobacterium avium* subsp. *paratuberculosis*, a pathogen of particular importance for adult sheep and goats [22]. On the other hand, using this technology, it has become possible to detect pathogens, which would not normally be diagnosed under a standardized array of laboratory methods exclusively for sheep and goat samples, for example, *P. shigelloides*.

Previously, Buss et al. [7] evaluated the technology in a collection of 1556 faecal samples, by comparing its findings against results obtained by conventional faecal cultures and molecular methods. FilmArray^®^ GI Panel had >97% specificity for all panel targets, while its sensitivity was found to be 100% for *P. shigelloides*, *Salmonella* spp., *Y. enterocolitica*, enterotoxigenic *E. coli* lt/st, *E. coli* O157, *Cryptosporidium* spp., *C. cayetanensis*, *G. lamblia*, Astrovirus, Rotavirus A and Sapovirus and 95% for *Campylobacter* spp., *C. difficile* (toxin A/B), enteroaggregative *E. coli*, enteropathogenic *E. coli*, *Shigella*/enteroinvasive *E. coli*, Adenovirus F 40/41 and Norovirus.

The increased sensitivity of the technology was confirmed by comparing the present results to those of Lianou et al. [20], who, using conventional parasitological techniques, declared significantly fewer farms to be ‘positive’. Conventional methods used to detect protozoan parasites in faecal samples lack sensitivity compared to molecular tests. FilmArray^®^ GI did not differentiate between live pathogens or genetic material, while conventional techniques could detect only parasites, i.e., were able to find fewer, positive farms, but not necessarily ones with active infections. Antigen detection tests are more sensitive and less laborious than microscopic observation. In the present study, the results do not refer exclusively to active infections, and this also became evident from the limited adverse effects in the animals sampled, which referred only to a lower body condition score, with no reduction in milk production.

With regard to *P. shigelloides*, it is noteworthy that this is the first report of detection of the pathogen from small ruminants. The organism is a confirmed pathogen of aquatic environments [23,24]. Therefore, we postulate that possibly animals became infected when they drank water from water enclosures during grazing.

Possibly, the technology as is can be employed particularly in samples from sheep or goats for the specific detection of pathogens of zoonotic importance. Nevertheless, the ease of use and the quick reporting of results point out to the usefulness of developing relevant veterinary tests, potentially species-specific.

### 4.2. Zoonotic Significance of Findings

There is a scarcity of data regarding the etiological agents of gastroenteritis in humans in Greece, as provided by the National Public Health Organization (NPHO) of the country [25], and hence there are few possibilities to make inferences regarding the significance of findings for human infections. Nevertheless, there is scope to consider the findings from a zoonotic viewpoint, given the widespread consumption of products of sheep or goat origin in Greece and the use of this human-oriented technology for the detection of pathogens.

For example, there is a difference between the high detection rate of enterohaemorrhagic *E. coli* from sheep and goat samples (approximately 90%) and the very low incidence in people in the country (reported as ~0.4 cases per million annually in 2019 and 2020 [25]). First, this indicates the quality standards for controls and hygiene procedures throughout the chain from the farm to the consumers. Moreover, it should be indicated again that the clinical reports from the NPHO refer to active infections in humans, which would be in line with the lower frequency of such cases.

The NPHO has also reported that the frequency of *Salmonella* spp.-related cases of gastrointestinal infections in humans in the country was higher than that of *Campylobacter*-related cases [25]. This significant contrast to the present findings points out that for *Salmonella,* human infections from other foods (e.g., pork meat, eggs) should be kept in mind.

### 4.3. Variables Associated with Gastrointestinal Infections

The infections were associated with a detrimental effect in the body condition score of ewes. In adult sheep, bodyweight loss and deterioration of condition score are the usual adverse effects of gastrointestinal infections, with clinical signs being present only in the cases of high infection pressure. Nevertheless, ‘ill-thrift’ is of economic and welfare concern [26] and farmers must be cautious despite the lack of relevant clinical presentations.

The more frequent detection of *Salmonella* from sheep than from goats is in line with previous reports [27,28]. This can be attributed to differences in the grazing behavior between the two animal species, in particular feed selectivity. Sheep forage mainly grasses rather than shrubs (60% and 10%, respectively, of total forage intake), while goats forage mainly shrubs rather than grasses (60% and 20%, respectively, of total forage intake) [29,30]. This is the result of anatomical adaptations of the mouths of respective species, which permit the respective type of grazing [31]. All the above might have contributed in sheep ingesting *Salmonella* more easily than goats, resulting in more frequent infections of these animals.

The analysis of management-related factors did not provide many significant results. The identification of the association of detection of *Y. enterocolitica* with machine milking of sheep and goats is of concern, because it suggests that the pathogen may potentially be excreted during milking (i.e., when the animals are on a high ramp in the milking parlour, at which time small ruminants often defecate [32,33]). Hence, there is a risk of infection of the milkers who work at face level with the back of the animals being milked.

The association of the number of pathogens detected in samples with the climatic conditions in goat, but not sheep, farms could be the result of the generally more extensive type of management in this animal species (the most common type of management system in goat farms was found to be the semi-extensive, whilst, in contrast, in sheep farms the most common type of management system was found to be the semi-intensive). The positive association with the precipitation could be the consequence of pathogens benefiting and multiplying at a higher rate in a wet soil, hencehaving more chances to infect the grazing animals. Moreover, the inverse association with the temperature range could be the result of the destructive effect of a wide range of temperature on pathogens, as these may withstand small temperature differences, but not wider ones [34]. The relevant literature presents conflicting results regarding possible effects of soil temperature and humidity on pathogen communities [35]: for example, Smit et al. [36] and Liesack et al. [37] reported substantial effects, whereas Watanabe et al. [38] found that archaeal methanogenic community structure was largely unaffected by strong abiotic seasonal changes. In any case, it is noteworthy that a seasonal pattern was reported in people for gastrointestinal infections of foodborne origin [39].

## 5. Conclusions

FilmArray^®^ GI Panel was found useful for the detection of zoonotic gastrointestinal pathogens in small ruminants. Pathogens were found in all samples (prevalence: 100.0%), with Shiga-like toxin-producing *E. coli* stx1/stx2 detected in samples from most farms. Mixed infections caused an adverse effect in the body condition score of sheep. Climatic factors, specifically precipitation and temperature range, were found to be associated with the detection of higher number of pathogens in faecal samples from goats.

## Figures and Tables

**Table 1:** Prevalence (95% confidence intervals) of detection of gastrointestinal pathogens in small ruminant farms (*n* = 94) in Greece, as found by means of the BioFire^®^ FilmArray^®^ Gastrointestinal (GI) Panel multiplex-PCR.

Gastrointestinal Pathogens	Sheep Flocks (*n* = 70)	Goat Herds (*n* = 24)
*Campylobacter* (*jejuni*, *coli*, *upsaliensis*)	52.9% (41.3–64.1%)	41.7% (24.5–61.2%)
*Clostridium difficile* (toxin A/B)	0.0% (0.0–5.2%)	0.0% (0.0–13.8%)
*Plesiomonas shigelloides*	0.0% (0.0–5.2%)	4.2% (0.7–20.3%)
*Salmonella* spp.	18.6% (11.2–29.2%) *	0.0% (0.0–13.8%) *
*Vibrio* (*parahaemolyticus*, *vulnificus*, *cholerae*)	0.0% (0.0–5.2%)	0.0% (0.0–13.8%)
*Vibrio cholerae*	0.0% (0.0–5.2%)	0.0% (0.0–13.8%)
*Yersinia enterocolitica*	11.4% (5.9–21.0%)	12.5% (4.3–31.0%)
Enteroaggregative *Escherichia coli*	0.0% (0.0–5.2%)	0.0% (0.0–13.8%)
Enteropathogenic *E. coli*	0.0% (0.0–5.2%)	0.0% (0.0–13.8%)
Enterotoxigenic *E. coli* lt/st	11.4% (5.9–21.0%)	20.8% (9.2–40.5%)
Shiga-like toxin-producing *E. coli* stx1/stx2	97.1% (90.2–99.2%)	87.5% (69.0–95.7%)
*E. coli* O157	8.6% (4.0–17.5%)	16.7% (6.7–35.9%)
*Shigella*/enteroinvasive *E. coli*	2.9% (0.8–9.8%)	8.3% (2.2–25.8%)
*Cryptosporidium*	15.7% (9.0–26.0%)	12.5% (4.3–31.0%)
*Cyclospora cayetanensis*	0.0% (0.0–5.2%)	0.0% (0.0–13.8%)
*Entamoeba histolytica*	0.0% (0.0–5.2%)	0.0% (0.0–13.8%)
*Giardia lamblia*	60.0% (48.3–70.7%)	58.3% (38.8–75.5%)
Adenovirus F40/41	0.0% (0.0–5.2%)	0.0% (0.0–13.8%)
Astrovirus	0.0% (0.0–5.2%)	0.0% (0.0–13.8%)
Norovirus GI/GII,	0.0% (0.0–5.2%)	0.0% (0.0–13.8%)
Rotavirus A	4.3% (1.5–11.9%)	8.3% (2.2–25.8%)
Sapovirus (I, II, IV, and V)	0.0% (0.0–5.2%)	0.0% (0.0–13.8%)

* *p* = 0.022 for the difference in prevalence between sheep and goat farms.

**Table 2:** Results of significant associations (univariable analysis) of management-related variables with detection of specific gastrointestinal pathogens in faecal samples from sheep (*n* = 70) or goat (*n* = 24) farms in Greece, as found by means of the BioFire^®^ FilmArray^®^ Gastrointestinal (GI) Panel multiplex-PCR ^1^.

**Sheep Farms**								
Detection of *Yersinia enterocolitica* in faecal samples (*n* = 8)				No detection of *Y. enterocolitica* in faecal samples (*n* = 62)				
Management system applied on the farm								
Intensive (*n* = 8)	Semi intensive (*n* = 38)	Semi-extensive (*n* = 24)	Extensive (*n* = 0)	Intensive (*n* = 8)	Semi intensive (*n* = 38)	Semi-extensive (*n* = 24)	Extensive (*n* = 0)	
0	2	6	0	8	36	18	0	0.033
Availability of milking parlour								
Yes (*n* = 60)		No (*n* = 10)		Yes (*n* = 60)		No (*n* = 10)		
4		4		56		6		0.002
Detection of enterotoxigenic *Escherichia coli* lt/st in faecal samples (*n* = 8)				No detection of enterotoxigenic *E. coli* lt/st in faecal samples (*n* = 62)				
Availability of milking parlour								
Yes (*n* = 60)		No (*n* = 10)		Yes (*n* = 60)		No (*n* = 10)		
5		3		55		7		0.046
**Goat farms**								
Detection of enterotoxigenic *E. coli* lt/st in faecal samples (*n* = 5)				No detection of enterotoxigenic *E. coli* lt/st in faecal samples (*n* = 19)				
Availability of milking parlour								
Yes (*n* = 13)		No (*n* = 11)		Yes (*n* = 13)		No (*n* = 11)		
5		0		8		11		0.020

^1^ The results of the assessments for all variables and all pathogens are presented in detail in Tables S1 and S2.

## Data Availability

All relevant data are available in the Supplementary Material.

## Supplementary Materials

The following supporting information can be downloaded at: https://www.mdpi.com/article/10.3390/microorganisms10040714/s1, Table S1: Results of evaluation of the association of variables with detection of specific gastrointestinal pathogens in faecal samples from sheep farms (*n* = 70) in Greece, as found by means of the BioFire^®^ FilmArray^®^ Gastrointestinal (GI) Panel multiplex-PCR; Table S2: Results of evaluation of the association of variables with detection of specific gastrointestinal pathogens in in faecal samples from goat farms (*n* = 24) in Greece, as found by means of the BioFire^®^ FilmArray^®^ Gastrointestinal (GI) Panel multiplex-PCR; Table S3: Results of evaluation of the association of variables with the number of gastrointestinal pathogens in faecal samples from small ruminant farms in Greece, as found by means of the BioFire^®^ FilmArray^®^ Gastrointestinal (GI) Panel multiplex-PCR.
