# Supplementary material for: Detection of Zoonotic Gastrointestinal Pathogens in Dairy Sheep and Goats by Using FilmArray® Multiplex-PCR Technology
Source: Microorganisms. 2022 Mar 25;10(4):714. doi: 10.3390/microorganisms10040714 (PMC9030461; doi:10.3390/microorganisms10040714)
Supplement: Supplementary file 1 [file microorganisms-10-00714-s001.zip › microorganisms-1634419-supplementary.pdf]

Detection of Gastrointestinal Pathogens in Dairy Sheep and Goats by Using FilmArray® Multiplex-PCR Technology

Katerina Tsilipounidaki, Zoe Florou, Daphne T. Lianou, Charalambia K. Michael, Anargyros Skoulakis, George C. Fthenakis and Efthymia Petinaki

Table S1. Results of evaluation of the association of management-related variables with detection of specific gastrointestinal pathogens in faecal samples from sheep farms (*n* = 70) in Greece, as found by means of the BioFire® FilmArray® Gastrointestinal (GI) Panel multiplex-PCR.

(a) *Campylobacter* spp.

**Detection of pathogen in faecal samples (*n* = 37)**				**No detection of pathogen in faecal samples (*n* = 33)**				***p***
**Management system applied in the farm**								
Intensive (*n* = 8)	Semi-intensive (*n* = 38)	Semi-extensive (*n* = 24)	Extensive (*n* = 0)	Intensive (*n* = 8)	Semi-intensive (*n* = 38)	Semi-extensive (*n* = 24)	Extensive (*n* = 0)	
3	19	15	0	5	19	9	0	0.16
**Month into the lactation period at sampling**								
0 – 1st (n = 6)	2nd – 5th (n = 56)	6th – 9th (n = 8)	After 9th (n = 0)	0 – 1st (n = 6)	2nd – 5th (n = 56)	6th – 9th (n = 8)	After 9th (n = 0)	
2	31	4	0	4	25	4	0	0.88
**Availability of milking parlour**								
Yes (n = 60)		No (n = 10)		Yes (n = 60)		No (n = 10)		
31		6		29		4		0.32
**No. of ewes in the flock**								
≤ 165 ewes (n = 19)	166 - 330 ewes (n = 26)	331 - 500 ewes (n = 11)	> 500 ewes (n = 14)	≤ 165 ewes (n = 19)	166 - 330 ewes (n = 26)	331 - 500 ewes (n = 11)	> 500 ewes (n = 14)	
10	11	6	10	9	15	5	4	0.83

(b) *Salmonella* spp.

**Detection of pathogen in faecal samples (*n* = 13)**				**No detection of pathogen in faecal samples (*n* = 57)**				***p***
**Management system applied in the farm**								
Intensive (*n* = 8)	Semi-intensive (*n* = 38)	Semi-extensive (*n* = 24)	Extensive (*n* = 0)	Intensive (*n* = 8)	Semi-intensive (*n* = 38)	Semi-extensive (*n* = 24)	Extensive (*n* = 0)	
2	4	7	0	6	34	17	0	0.16
**Month into the lactation period at sampling**								
0 – 1st (n = 6)	2nd – 5th (n = 56)	6th – 9th (n = 8)	After 9th (n = 0)	0 – 1st (n = 6)	2nd – 5th (n = 56)	6th – 9th (n = 8)	After 9th (n = 0)	
1	11	1	0	5	45	7	0	0.88
**Availability of milking parlour**								
Yes (n = 60)		No (n = 10)		Yes (n = 60)		No (n = 10)		
10		3		50		7		0.32
**No. of ewes in the flock**								
≤ 165 ewes (n = 19)	166 - 330 ewes (n = 26)	331 - 500 ewes (n = 11)	> 500 ewes (n = 14)	≤ 165 ewes (n = 19)	166 - 330 ewes (n = 26)	331 - 500 ewes (n = 11)	> 500 ewes (n = 14)	
3	4	3	3	16	22	8	11	0.83

(c) *Yersinia enterocolitica*

**Detection of pathogen in faecal samples (*n* = 8)**				**No detection of pathogen in faecal samples (*n* = 62)**				***p***
**Management system applied in the farm**								
Intensive (*n* = 8)	Semi-intensive (*n* = 38)	Semi-extensive (*n* = 24)	Extensive (*n* = 0)	Intensive (*n* = 8)	Semi-intensive (*n* = 38)	Semi-extensive (*n* = 24)	Extensive (*n* = 0)	
0	2	6	0	8	36	18	0	0.033
**Month into the lactation period at sampling**								
0 – 1st (n = 6)	2nd – 5th (n = 56)	6th – 9th (n = 8)	After 9th (n = 0)	0 – 1st (n = 6)	2nd – 5th (n = 56)	6th – 9th (n = 8)	After 9th (n = 0)	
0	7	1	0	6	49	7	0	0.65

**Availability of milking parlour**								
Yes (n = 60)		No (n = 10)		Yes (n = 60)		No (n = 10)		
4		4		56		6		0.002
**No. of ewes in the flock**								
≤ 165 ewes (n = 19)	166 - 330 ewes (n = 26)	331 - 500 ewes (n = 11)	> 500 ewes (n = 14)	≤ 165 ewes (n = 19)	166 - 330 ewes (n = 26)	331 - 500 ewes (n = 11)	> 500 ewes (n = 14)	
2	4	1	1	17	22	10	13	0.83

(d) Enterotoxigenic *E. coli* lt/st

**Detection of pathogen in faecal samples (*n* = 8)**				**No detection of pathogen in faecal samples (*n* = 62)**				***p***
**Management system applied in the farm**								
Intensive (*n* = 8)	Semi-intensive (*n* = 38)	Semi-extensive (*n* = 24)	Extensive (*n* = 0)	Intensive (*n* = 8)	Semi-intensive (*n* = 38)	Semi-extensive (*n* = 24)	Extensive (*n* = 0)	
0	3	5	0	8	35	19	0	0.16
**Month into the lactation period at sampling**								
0 – 1st (n = 6)	2nd – 5th (n = 56)	6th – 9th (n = 8)	After 9th (n = 0)	0 – 1st (n = 6)	2nd – 5th (n = 56)	6th – 9th (n = 8)	After 9th (n = 0)	
1	7	0	0	5	49	8	0	0.53
**Availability of milking parlour**								
Yes (n = 60)		No (n = 10)		Yes (n = 60)		No (n = 10)		
5		3		55		7		0.046
**No. of ewes in the flock**								
≤ 165 ewes (n = 19)	166 - 330 ewes (n = 26)	331 - 500 ewes (n = 11)	> 500 ewes (n = 14)	≤ 165 ewes (n = 19)	166 - 330 ewes (n = 26)	331 - 500 ewes (n = 11)	> 500 ewes (n = 14)	
1	5	1	1	18	21	10	13	0.46

(e) Shiga-like toxin-producing *E. coli* stx1/stx2

**Detection of pathogen in faecal samples (*n* = 68)**				**No detection of pathogen in faecal samples (*n* = 2)**				***p***
**Management system applied in the farm**								
Intensive (*n* = 8)	Semi-intensive (*n* = 38)	Semi-extensive (*n* = 24)	Extensive (*n* = 0)	Intensive (*n* = 8)	Semi-intensive (*n* = 38)	Semi-extensive (*n* = 24)	Extensive (*n* = 0)	
8	36	24	0	0	2	0	0	0.42
**Month into the lactation period at sampling**								
0 – 1st (n = 6)	2nd – 5th (n = 56)	6th – 9th (n = 8)	After 9th (n = 0)	0 – 1st (n = 6)	2nd – 5th (n = 56)	6th – 9th (n = 8)	After 9th (n = 0)	
6	54	8	0	0	2	0	0	0.77
**Availability of milking parlour**								
Yes (n = 60)		No (n = 10)		Yes (n = 60)		No (n = 10)		
58		10		2		0		0.56
**No. of ewes in the flock**								
≤ 165 ewes (n = 19)	166 - 330 ewes (n = 26)	331 - 500 ewes (n = 11)	> 500 ewes (n = 14)	≤ 165 ewes (n = 19)	166 - 330 ewes (n = 26)	331 - 500 ewes (n = 11)	> 500 ewes (n = 14)	
19	24	11	14	0	2	0	0	0.32

(f) *E. coli* O157

**Detection of pathogen in faecal samples (*n* = 6)**				**No detection of pathogen in faecal samples (*n* = 64)**				***p***
**Management system applied in the farm**								
Intensive (*n* = 8)	Semi-intensive (*n* = 38)	Semi-extensive (*n* = 24)	Extensive (*n* = 0)	Intensive (*n* = 8)	Semi-intensive (*n* = 38)	Semi-extensive (*n* = 24)	Extensive (*n* = 0)	
1	1	4	0	7	37	20	0	0.14
**Month into the lactation period at sampling**								
0 – 1st (n = 6)	2nd – 5th (n = 56)	6th – 9th (n = 8)	After 9th (n = 0)	0 – 1st (n = 6)	2nd – 5th (n = 56)	6th – 9th (n = 8)	After 9th (n = 0)	
1	5	0	0	5	51	8	0	0.53

**Availability of milking parlour**								
Yes (n = 60)		No (n = 10)		Yes (n = 60)		No (n = 10)		
4		2		56		8		0.16
**No. of ewes in the flock**								
≤ 165 ewes (n = 19)	166 - 330 ewes (n = 26)	331 - 500 ewes (n = 11)	> 500 ewes (n = 14)	≤ 165 ewes (n = 19)	166 - 330 ewes (n = 26)	331 - 500 ewes (n = 11)	> 500 ewes (n = 14)	
0	3	2	1	19	23	9	13	0.33

(g) *Shigella* / enteroinvasive *E. coli*

**Detection of pathogen in faecal samples (*n* = 2)**				**No detection of pathogen in faecal samples (*n* = 68)**				***p***
**Management system applied in the farm**								
Intensive (*n* = 8)	Semi-intensive (*n* = 38)	Semi-extensive (*n* = 24)	Extensive (*n* = 0)	Intensive (*n* = 8)	Semi-intensive (*n* = 38)	Semi-extensive (*n* = 24)	Extensive (*n* = 0)	
0	1	1	0	8	37	23	0	0.82
**Month into the lactation period at sampling**								
0 – 1st (n = 6)	2nd – 5th (n = 56)	6th – 9th (n = 8)	After 9th (n = 0)	0 – 1st (n = 6)	2nd – 5th (n = 56)	6th – 9th (n = 8)	After 9th (n = 0)	
0	2	0	0	6	54	8	0	0.77
**Availability of milking parlour**								
Yes (n = 60)		No (n = 10)		Yes (n = 60)		No (n = 10)		
2		0		58		10		0.56
**No. of ewes in the flock**								
≤ 165 ewes (n = 19)	166 - 330 ewes (n = 26)	331 - 500 ewes (n = 11)	> 500 ewes (n = 14)	≤ 165 ewes (n = 19)	166 - 330 ewes (n = 26)	331 - 500 ewes (n = 11)	> 500 ewes (n = 14)	
0	1	0	1	19	25	11	13	0.59

(h) *Cryptosporidium* spp.

Detection of pathogen in faecal samples (*n* = 11)				No detection of pathogen in faecal samples (*n* = 59)				*p*
**Management system applied in the farm**								
Intensive (*n* = 8)	Semi-intensive (*n* = 38)	Semi-extensive (*n* = 24)	Extensive (*n* = 0)	Intensive (*n* = 8)	Semi-intensive (*n* = 38)	Semi-extensive (*n* = 24)	Extensive (*n* = 0)	
0	4	7	0	8	34	17	0	0.06
**Month into the lactation period at sampling**								
0 – 1st (n = 6)	2nd – 5th (n = 56)	6th – 9th (n = 8)	After 9th (n = 0)	0 – 1st (n = 6)	2nd – 5th (n = 56)	6th – 9th (n = 8)	After 9th (n = 0)	
2	7	2	0	4	49	6	0	0.31
**Availability of milking parlour**								
Yes (n = 60)		No (n = 10)		Yes (n = 60)		No (n = 10)		
9		2		51		8		0.69
**No. of ewes in the flock**								
≤ 165 ewes (n = 19)	166 - 330 ewes (n = 26)	331 - 500 ewes (n = 11)	> 500 ewes (n = 14)	≤ 165 ewes (n = 19)	166 - 330 ewes (n = 26)	331 - 500 ewes (n = 11)	> 500 ewes (n = 14)	
4	4	2	1	15	22	9	13	0.74

(i) *Lamblia giardia*

Detection of pathogen in faecal samples (*n* = 42)				No detection of pathogen in faecal samples (*n* = 28)				*p*
**Management system applied in the farm**								
Intensive (*n* = 8)	Semi-intensive (*n* = 38)	Semi-extensive (*n* = 24)	Extensive (*n* = 0)	Intensive (*n* = 8)	Semi-intensive (*n* = 38)	Semi-extensive (*n* = 24)	Extensive (*n* = 0)	
5	21	16	0	3	17	8	0	0.66
**Month into the lactation period at sampling**								
0 – 1st (n = 6)	2nd – 5th (n = 56)	6th – 9th (n = 8)	After 9th (n = 0)	0 – 1st (n = 6)	2nd – 5th (n = 56)	6th – 9th (n = 8)	After 9th (n = 0)	
4	34	4	0	2	22	4	0	0.80

**Availability of milking parlour**								
Yes (n = 60)		No (n = 10)		Yes (n = 60)		No (n = 10)		
35		7		25		3		0.49
**No. of ewes in the flock**								
≤ 165 ewes (n = 19)	166 - 330 ewes (n = 26)	331 - 500 ewes (n = 11)	> 500 ewes (n = 14)	≤ 165 ewes (n = 19)	166 - 330 ewes (n = 26)	331 - 500 ewes (n = 11)	> 500 ewes (n = 14)	
8	17	7	10	11	9	4	4	0.30

(j) Rotavirus A

**Detection of pathogen in faecal samples (*n* = 3)**				**No detection of pathogen in faecal samples (*n* = 67)**				***p***
**Management system applied in the farm**								
Intensive (*n* = 8)	Semi-intensive (*n* = 38)	Semi-extensive (*n* = 24)	Extensive (*n* = 0)	Intensive (*n* = 8)	Semi-intensive (*n* = 38)	Semi-extensive (*n* = 24)	Extensive (*n* = 0)	
0	1	2	0	8	37	22	0	0.46
**Month into the lactation period at sampling**								
0 – 1st (n = 6)	2nd – 5th (n = 56)	6th – 9th (n = 8)	After 9th (n = 0)	0 – 1st (n = 6)	2nd – 5th (n = 56)	6th – 9th (n = 8)	After 9th (n = 0)	
0	2	1	0	6	54	7	0	0.44
**Availability of milking parlour**								
Yes (n = 60)		No (n = 10)		Yes (n = 60)		No (n = 10)		
3		0		57		10		0.47
**No. of ewes in the flock**								
≤ 165 ewes (n = 19)	166 - 330 ewes (n = 26)	331 - 500 ewes (n = 11)	> 500 ewes (n = 14)	≤ 165 ewes (n = 19)	166 - 330 ewes (n = 26)	331 - 500 ewes (n = 11)	> 500 ewes (n = 14)	
2	0	0	1	17	26	11	13	0.29

Table S2. Results of evaluation of the association of management-related variables with detection of specific gastrointestinal pathogens in faecal samples from goat farms (*n* = 24) in Greece, as found by means of the BioFire® FilmArray® Gastrointestinal (GI) Panel multiplex-PCR.

(a) *Campylobacter* spp.

**Detection of pathogen in faecal samples (*n* = 10)**				**No detection of pathogen in faecal samples (*n* = 14)**				***p***
**Management system applied in the farm**								
Intensive (*n* = 4)	Semi-intensive (*n* = 5)	Semi-extensive (*n* = 10)	Extensive (*n* = 5)	Intensive (*n* = 4)	Semi-intensive (*n* = 5)	Semi-extensive (*n* = 10)	Extensive (*n* = 5)	
1	4	4	1	3	1	6	4	0.22
**Month into the lactation period at sampling**								
0 – 1st (n = 4)	2nd – 5th (n = 18)	6th – 9th (n = 1)	After 9th (n = 1)	0 – 1st (n = 4)	2nd – 5th (n = 18)	6th – 9th (n = 1)	After 9th (n = 1)	
3	7	0	0	1	11	1	1	0.35
**Availability of milking parlour**								
Yes (n = 13)		No (n = 11)		Yes (n = 13)		No (n = 11)		
7		3		6		8		0.19
**No. of does in the herd**								
≤ 165 does (n = 9)	166 - 330 does (n = 11)	331 - 500 does (n = 2)	> 500 does (n = 2)	≤ 165 does (n = 9)	166 - 330 does (n = 11)	331 - 500 does (n = 2)	> 500 does (n = 2)	
4	4	1	1	5	7	1	1	0.97

(b) *Yersinia enterocolitica*

**Detection of pathogen in faecal samples (*n* = 3)**				**No detection of pathogen in faecal samples (*n* = 21)**				***p***
**Management system applied in the farm**								
Intensive (*n* = 4)	Semi-intensive (*n* = 5)	Semi-extensive (*n* = 10)	Extensive (*n* = 5)	Intensive (*n* = 4)	Semi-intensive (*n* = 5)	Semi-extensive (*n* = 10)	Extensive (*n* = 5)	
0	0	2	1	4	5	8	4	0.56

**Month into the lactation period at sampling**								
0 – 1st (n = 4)	2nd – 5th (n = 18)	6th – 9th (n = 1)	After 9th (n = 1)	0 – 1st (n = 4)	2nd – 5th (n = 18)	6th – 9th (n = 1)	After 9th (n = 1)	
0	3	0	0	4	15	1	1	0.77
**Availability of milking parlour**								
Yes (n = 13)		No (n = 11)		Yes (n = 13)		No (n = 11)		
2		1		11		10		0.64
**No. of does in the herd**								
≤ 165 does (n = 9)	166 - 330 does (n = 11)	331 - 500 does (n = 2)	> 500 does (n = 2)	≤ 165 does (n = 9)	166 - 330 does (n = 11)	331 - 500 does (n = 2)	> 500 does (n = 2)	
0	2	1	0	9	9	1	2	0.22

(c) Enterotoxigenic *E. coli* lt/st

**Detection of pathogen in faecal samples (*n* = 5)**				**No detection of pathogen in faecal samples (*n* = 19)**				***p***
**Management system applied in the farm**								
Intensive (*n* = 4)	Semi-intensive (*n* = 5)	Semi-extensive (*n* = 10)	Extensive (*n* = 5)	Intensive (*n* = 4)	Semi-intensive (*n* = 5)	Semi-extensive (*n* = 10)	Extensive (*n* = 5)	
0	2	3	0	4	3	7	5	0.26
**Month into the lactation period at sampling**								
0 – 1st (n = 4)	2nd – 5th (n = 18)	6th – 9th (n = 1)	After 9th (n = 1)	0 – 1st (n = 4)	2nd – 5th (n = 18)	6th – 9th (n = 1)	After 9th (n = 1)	
0	5	0	0	4	13	1	1	0.55
**Availability of milking parlour**								
Yes (n = 13)		No (n = 11)		Yes (n = 13)		No (n = 11)		
5		0		8		11		0.020
**No. of does in the herd**								
≤ 165 does (n = 9)	166 - 330 does (n = 11)	331 - 500 does (n = 2)	> 500 does (n = 2)	≤ 165 does (n = 9)	166 - 330 does (n = 11)	331 - 500 does (n = 2)	> 500 does (n = 2)	
2	3	0	0	7	8	2	2	0.72

(d) Shiga-like toxin-producing *E. coli* stx1/stx2

Detection of pathogen in faecal samples (*n* = 21)				No detection of pathogen in faecal samples (*n* = 3)				*p*
**Management system applied in the farm**								
Intensive (*n* = 4)	Semi-intensive (*n* = 5)	Semi-extensive (*n* = 10)	Extensive (*n* = 5)	Intensive (*n* = 4)	Semi-intensive (*n* = 5)	Semi-extensive (*n* = 10)	Extensive (*n* = 5)	
4	5	9	3	0	0	1	2	0.19
**Month into the lactation period at sampling**								
0 – 1st (n = 4)	2nd – 5th (n = 18)	6th – 9th (n = 1)	After 9th (n = 1)	0 – 1st (n = 4)	2nd – 5th (n = 18)	6th – 9th (n = 1)	After 9th (n = 1)	
4	15	1	1	0	3	0	0	0.77
**Availability of milking parlour**								
Yes (n = 13)		No (n = 11)		Yes (n = 13)		No (n = 11)		
12		9		1		2		0.44
**No. of does in the herd**								
≤ 165 does (n = 9)	166 - 330 does (n = 11)	331 - 500 does (n = 2)	> 500 does (n = 2)	≤ 165 does (n = 9)	166 - 330 does (n = 11)	331 - 500 does (n = 2)	> 500 does (n = 2)	
8	9	2	2	1	2	0	0	0.82

(e) *E. coli* O157

Detection of pathogen in faecal samples (*n* = 4)				No detection of pathogen in faecal samples (*n* = 20)				*p*
**Management system applied in the farm**								
Intensive (*n* = 4)	Semi-intensive (*n* = 5)	Semi-extensive (*n* = 10)	Extensive (*n* = 5)	Intensive (*n* = 4)	Semi-intensive (*n* = 5)	Semi-extensive (*n* = 10)	Extensive (*n* = 5)	
2	1	0	1	2	4	10	4	0.15
**Month into the lactation period at sampling**								
0 – 1st (n = 4)	2nd – 5th (n = 18)	6th – 9th (n = 1)	After 9th (n = 1)	0 – 1st (n = 4)	2nd – 5th (n = 18)	6th – 9th (n = 1)	After 9th (n = 1)	
0	4	0	0	4	14	1	1	0.66

**Availability of milking parlour**								
Yes (n = 13)		No (n = 11)		Yes (n = 13)		No (n = 11)		
1		3		12		8		0.20
**No. of does in the herd**								
≤ 165 does (n = 9)	166 - 330 does (n = 11)	331 - 500 does (n = 2)	> 500 does (n = 2)	≤ 165 does (n = 9)	166 - 330 does (n = 11)	331 - 500 does (n = 2)	> 500 does (n = 2)	
2	1	0	1	7	10	2	1	0.45

(f) *Shigella* / enteroinvasive *E. coli*

**Detection of pathogen in faecal samples (*n* = 2)**				**No detection of pathogen in faecal samples (*n* = 22)**				***p***
**Management system applied in the farm**								
Intensive (*n* = 4)	Semi-intensive (*n* = 5)	Semi-extensive (*n* = 10)	Extensive (*n* = 5)	Intensive (*n* = 4)	Semi-intensive (*n* = 5)	Semi-extensive (*n* = 10)	Extensive (*n* = 5)	
0	0	1	1	4	5	9	4	0.63
**Month into the lactation period at sampling**								
0 – 1st (n = 4)	2nd – 5th (n = 18)	6th – 9th (n = 1)	After 9th (n = 1)	0 – 1st (n = 4)	2nd – 5th (n = 18)	6th – 9th (n = 1)	After 9th (n = 1)	
0	2	0	0	4	16	1	1	0.87
**Availability of milking parlour**								
Yes (n = 13)		No (n = 11)		Yes (n = 13)		No (n = 11)		
1		1		12		10		0.90
**No. of does in the herd**								
≤ 165 does (n = 9)	166 - 330 does (n = 11)	331 - 500 does (n = 2)	> 500 does (n = 2)	≤ 165 does (n = 9)	166 - 330 does (n = 11)	331 - 500 does (n = 2)	> 500 does (n = 2)	
0	2	0	0	9	9	2	2	0.46

(g) *Cryptosporidium* spp.

Detection of pathogen in faecal samples (*n* = 3)				No detection of pathogen in faecal samples (*n* = 21)				*p*
**Management system applied in the farm**								
Intensive (*n* = 4)	Semi-intensive (*n* = 5)	Semi-extensive (*n* = 10)	Extensive (*n* = 5)	Intensive (*n* = 4)	Semi-intensive (*n* = 5)	Semi-extensive (*n* = 10)	Extensive (*n* = 5)	
0	1	2	0	4	4	8	5	0.56
**Month into the lactation period at sampling**								
0 – 1st (n = 4)	2nd – 5th (n = 18)	6th – 9th (n = 1)	After 9th (n = 1)	0 – 1st (n = 4)	2nd – 5th (n = 18)	6th – 9th (n = 1)	After 9th (n = 1)	
1	2	0	0	3	16	1	1	0.83
**Availability of milking parlour**								
Yes (n = 13)		No (n = 11)		Yes (n = 13)		No (n = 11)		
2		1		11		10		0.64
**No. of does in the herd**								
≤ 165 does (n = 9)	166 - 330 does (n = 11)	331 - 500 does (n = 2)	> 500 does (n = 2)	≤ 165 does (n = 9)	166 - 330 does (n = 11)	331 - 500 does (n = 2)	> 500 does (n = 2)	
0	3	0	0	9	8	2	2	0.26

(h) *Lamblia giardia*

Detection of pathogen in faecal samples (*n* = 14)				No detection of pathogen in faecal samples (*n* = 10)				*p*
**Management system applied in the farm**								
Intensive (*n* = 4)	Semi-intensive (*n* = 5)	Semi-extensive (*n* = 10)	Extensive (*n* = 5)	Intensive (*n* = 4)	Semi-intensive (*n* = 5)	Semi-extensive (*n* = 10)	Extensive (*n* = 5)	
2	2	6	4	2	3	4	1	0.62
**Month into the lactation period at sampling**								
0 – 1st (n = 4)	2nd – 5th (n = 18)	6th – 9th (n = 1)	After 9th (n = 1)	0 – 1st (n = 4)	2nd – 5th (n = 18)	6th – 9th (n = 1)	After 9th (n = 1)	
3	11	0	0	1	7	1	1	0.35

**Availability of milking parlour**								
Yes (n = 13)		No (n = 11)		Yes (n = 13)		No (n = 11)		
7		7		6		4		0.63
**No. of does in the herd**								
≤ 165 does (n = 9)	166 - 330 does (n = 11)	331 - 500 does (n = 2)	> 500 does (n = 2)	≤ 165 does (n = 9)	166 - 330 does (n = 11)	331 - 500 does (n = 2)	> 500 does (n = 2)	
4	7	1	2	5	4	1	0	0.51

(i) Rotavirus A

**Detection of pathogen in faecal samples (*n* = 2)**				**No detection of pathogen in faecal samples (*n* = 22)**				***p***
**Management system applied in the farm**								
Intensive (*n* = 4)	Semi-intensive (*n* = 5)	Semi-extensive (*n* = 10)	Extensive (*n* = 5)	Intensive (*n* = 4)	Semi-intensive (*n* = 5)	Semi-extensive (*n* = 10)	Extensive (*n* = 5)	
0	0	1	1	4	5	9	4	0.63
**Month into the lactation period at sampling**								
0 – 1st (n = 4)	2nd – 5th (n = 18)	6th – 9th (n = 1)	After 9th (n = 1)	0 – 1st (n = 4)	2nd – 5th (n = 18)	6th – 9th (n = 1)	After 9th (n = 1)	
0	2	0	0	4	16	1	1	0.87
**Availability of milking parlour**								
Yes (n = 13)		No (n = 11)		Yes (n = 13)		No (n = 11)		
3		0		57		10		0.11
**No. of does in the herd**								
≤ 165 does (n = 9)	166 - 330 does (n = 11)	331 - 500 does (n = 2)	> 500 does (n = 2)	≤ 165 does (n = 9)	166 - 330 does (n = 11)	331 - 500 does (n = 2)	> 500 does (n = 2)	
0	2	0	0	9	9	2	2	0.46

Table S3. Results of evaluation of the association of management-related variables with the number of gastrointestinal pathogens in faecal samples from small ruminant farms in Greece, as found by means of the BioFire® FilmArray® Gastrointestinal (GI) Panel multiplex-PCR.

(a) Sheep flocks (*n* = 70)

**Management system applied in the farm**				
Intensive (*n* = 8)	Semi-intensive (*n* = 38)	Semi-extensive (*n* = 24)	Extensive (*n* = 0)	*p*
2.3	2.3	3.0	n/a	0.010
**Month into the lactation period at sampling**				
0 – 1st (n = 6)	2nd – 5th (n = 56)	6th – 9th (n = 8)	After 9th (n = 0)	*p*
2.5	2.6	2.6	n/a	0.97
**Availability of milking parlour**				
Yes (n = 60)		No (n = 10)		*p*
2.5		3.2		0.10
**No. of ewes in the flock**				
≤ 165 ewes (n = 19)	166 - 330 ewes (n = 26)	331 - 500 ewes (n = 11)	> 500 ewes (n = 14)	*p*
2.5	2.5	2.7	2.9	0.81

(b) Goat herds (*n* = 24)

**Management system applied in the farm**				
Intensive (*n* = 4)	Semi-intensive (*n* = 5)	Semi-extensive (*n* = 10)	Extensive (*n* = 5)	*p*
1.8	2.4	2.4	2.4	0.78
**Month into the lactation period at sampling**				
0 – 1st (n = 4)	2nd – 5th (n = 18)	6th – 9th (n = 1)	After 9th (n = 1)	*p*
2.8	2.3	1.0	1.0	0.49
**Availability of milking parlour**				
Yes (n = 13)		No (n = 11)		*p*
2.3		2.3		0.86

No. of does in the herd				
≤ 165 does (n = 9)	166 - 330 does (n = 11)	331 - 500 does (n = 2)	> 500 does (n = 2)	*p*
1.8	2.5	3.0	2.5	0.33

Table S4. Results of evaluation of the association of climate-related variables with the number of gastrointestinal pathogens in faecal samples from small ruminant farms in Greece, as found by means of the BioFire® FilmArray® Gastrointestinal (GI) Panel multiplex-PCR.

(a) Sheep flocks (*n* = 70)

**Average mean temperature at 2 m**			
Detection of 1 pathogen	Detection of 2 pathogens	Detection of ≥ 3 pathogens	*p*
6.6 ± 0.4 °C	5.5± 0.7 °C	6.5 ± 0.6 °C	0.52
**Average Earth skin temperature at 2 m**			
Detection of 1 pathogen	Detection of 2 pathogens	Detection of ≥ 3 pathogens	*p*
4.8 ± 0.8 °C	4.2± 0.9 °C	5.4 ± 0.8 °C	0.55
**Average minimum temperature at 2 m**			
Detection of 1 pathogen	Detection of 2 pathogens	Detection of ≥ 3 pathogens	*p*
2.8 ± 0.5 °C	1.9± 0.8 °C	3.0 ± 0.7 °C	0.53
**Average maximum temperature at 2 m**			
Detection of 1 pathogen	Detection of 2 pathogens	Detection of ≥ 3 pathogens	*p*
11.8 ± 0.3 °C	10.6± 0.6 °C	11.2 ± 0.5 °C	0.41
**Average temperature range at 2 m**			
Detection of 1 pathogen	Detection of 2 pathogens	Detection of ≥ 3 pathogens	*p*
9.0 ± 0.3 °C	8.7± 0.3 °C	8.2 ± 0.3 °C	0.21
**Average precipitation**			
Detection of 1 pathogen	Detection of 2 pathogens	Detection of ≥ 3 pathogens	*p*
1.29 ± 0.32 mm	1.64 ± 0.43 mm	2.06 ± 0.34 mm	0.42
**Average all sky insolation incident**			
Detection of 1 pathogen	Detection of 2 pathogens	Detection of ≥ 3 pathogens	p
6.3 ± 1.3 Wh m^{-2}	7.0 ± 0.7 Wh m^{-2}	7.2 ± 0.5 Wh m^{-2}	0.73

(b) Goat herds ($n = 24$)

**Average mean temperature at 2 m**			
Detection of 1 pathogen	Detection of 2 pathogens	Detection of ≥ 3 pathogens	*p*
3.7 ± 1.0 °C	6.1± 0.7 °C	6.4 ± 1.4 °C	0.24
**Average Earth skin temperature at 2 m**			
Detection of 1 pathogen	Detection of 2 pathogens	Detection of ≥ 3 pathogens	*p*
2.8 ± 1.3 °C	5.7± 0.8 °C	5.9 ± 1.7 °C	0.25
**Average minimum temperature at 2 m**			
Detection of 1 pathogen	Detection of 2 pathogens	Detection of ≥ 3 pathogens	*p*
–0.1 ± 0.9 °C	2.6± 0.8 °C	3.5 ± 1.7 °C	0.19
**Average maximum temperature at 2 m**			
Detection of 1 pathogen	Detection of 2 pathogens	Detection of ≥ 3 pathogens	*p*
9.0 ± 1.0 °C	10.6± 0.6 °C	10.5 ± 1.1 °C	0.30
**Average temperature range at 2 m**			
Detection of 1 pathogen	Detection of 2 pathogens	Detection of ≥ 3 pathogens	*p*
9.1 ± 0.3 °C	8.3± 0.4 °C	7.0 ± 0.7 °C	0.05
**Average precipitation**			
Detection of 1 pathogen	Detection of 2 pathogens	Detection of ≥ 3 pathogens	*p*
0.51 ± 0.20 mm	2.48 ± 0.60 mm	3.90 ± 1.18 mm	0.044
**Average all sky insolation incident**			
Detection of 1 pathogen	Detection of 2 pathogens	Detection of ≥ 3 pathogens	*p*
10.6 ± 0.4 Wh m^{-2}	9.1 ± 0.5 Wh m^{-2}	7.5 ± 0.6 Wh m^{-2}	0.07
